# Understanding of Colistin Usage in Food Animals and Available Detection Techniques: A Review

**DOI:** 10.3390/ani10101892

**Published:** 2020-10-16

**Authors:** Harsh Kumar, Bing-Huei Chen, Kamil Kuca, Eugenie Nepovimova, Ankur Kaushal, Rupak Nagraik, Shashi Kant Bhatia, Daljeet Singh Dhanjal, Vinod Kumar, Anil Kumar, Navneet Kumar Upadhyay, Rachna Verma, Dinesh Kumar

**Affiliations:** 1 School of Bioengineering & Food Technology, Shoolini University of Biotechnology and Management Sciences, Solan 173229, H.P., India; microharshs@gmail.com (H.K.); rupak.nagraik@gmail.com (R.N.); kumaranil@shooliniuniversity.com (A.K.); 2 Department of Food Science, Fu Jen Catholic University, New Taipei City 24205, Taiwan; 002622@mail.fju.edu.tw; 3 Department of Chemistry, Faculty of Science, University of Hradec Kralove, 50003 Hradec Kralove, Czech Republic; eugenie.nepovimova@uhk.cz; 4 Biomedical Research Center, University Hospital Hradec Kralove, 50003 Hradec Kralove, Czech Republic; 5 Centre of Nanotechnology, Amity University, Manesar, Gurugram-122413, Haryana, India; ankur.biotech85@gmail.com; 6 Department of Biological Engineering, College of Engineering, Konkuk University, Seoul 05029, Korea; shashikonkukuni@konkuk.ac.kr; 7 School of Bioengineering and Biosciences, Lovely Professional University, Phagwara 144411, Punjab, India; daljeetdhanjal92@gmail.com; 8 School of Water, Energy and Environment, Cranfield University, Cranfield MK430AL, UK; Vinod.Kumar@cranfield.ac.uk; 9 School of Pharmaceutical Sciences, Shoolini University of Biotechnology and Management Sciences, Solan 173229, H.P., India; navneetqa@gmail.com; 10 School of Biological and Environmental Sciences, Shoolini University of Biotechnology and Management Sciences, Solan 173229, H.P., India; rachnaverma@shooliniuniversity.com

**Keywords:** antibiotics, colistin, detection methods, food animals, multi-drug resistance

## Abstract

**Simple Summary:**

Colistin is a last resort drug for the treatment of infection caused by multidrug-resistant Gram-negative bacteria. Different studies have uncovered the negative impact of colistin consumption in animals. Therefore, it has become essential to monitor the dosing regimens of colistin and assess their negative effects. The current review intends to provide brief information of colistin usage and its associated negative impact and discuss available techniques to detect colistin in animal-based food so that effective preventive measures can be taken to minimize the health risks in both animals and humans.

**Abstract:**

Progress in the medical profession is determined by the achievements and effectiveness of new antibiotics in the treatment of microbial infections. However, the development of multiple-drug resistance in numerous bacteria, especially Gram-negative bacteria, has limited the treatment options. Due to this resistance, the resurgence of cyclic polypeptide drugs like colistin remains the only option. The drug, colistin, is a well-known growth inhibitor of Gram-negative bacteria like *Acinetobacter baumanni*, *Enterobacter cloacae*, *Klebsiella pneumoniae*, and *Pseudomonas aeruginosa.* Technological advancements have uncovered the role of the *mcr-1*(mobilized colistin resistance) gene, which is responsible for the development of resistance in Gram-negative bacteria, which make them distinct from other bacteria without this gene. Additionally, food animals have been determined to be the reservoir for colistin resistance microbes, from which they spread to other hosts. Due to the adverse effects of colistin, many developed countries have prohibited its usage in animal foods, but developing countries are still using colistin in animal food production, thereby imposing a major risk to the public health. Therefore, there is a need for implementation of sustainable measures in livestock farms to prevent microbial infection. This review highlights the negative effects (increased resistance) of colistin consumption and emphasizes the different approaches used for detecting colistin in animal-based foods as well as the challenges associated with its detection.

## 1. Introduction

**Figure 1:**
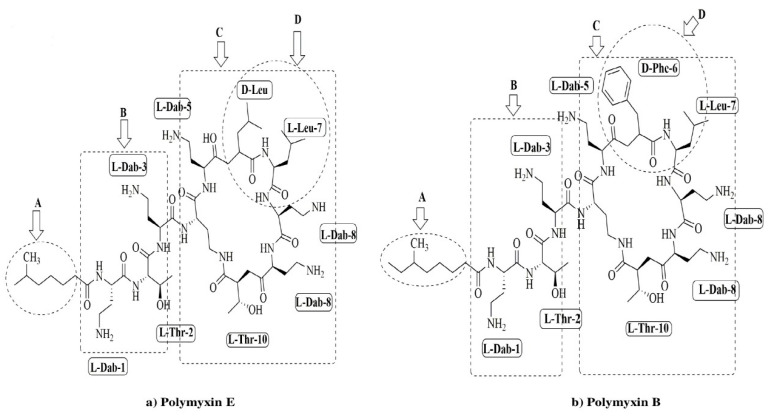
Chemical structures of polymyxin E (colistin) (**a**) and polymyxin B (**b**). The functional segments of polymyxins A: NR fatty acyl chain, B: linear tripeptide segment, C: the polar residues of the heptapeptide, D: the hydrophobic motif within the heptapeptide ring.

Colistin is an antibiotic synthesized non-ribosomally by *Bacillus polymyxa* subspecies *colistinus* [1]. Colistin (polymyxin E) and polymyxin B (PMB) have high structural similarities and differ only at position six, where D-Leu is present in colistin, and D-Phe is present in PMB, as illustrated in Figure 1.

Colistin is effective against various Gram-negative bacteria such as *Acinetobacter baumanni*, *Enterobactercloacae*, *Klebsiella pneumonia*, and *Pseudomonas aeruginosa* [1,2]. The mechanism of action of colistin involves interactions with the outer membrane of the organism, especially lipopolysaccharide molecules, which causes displacement of calcium and magnesium ions and destabilizes the outer membrane. This destabilization of the outer membrane causes the leakage of cell content and leads to cell senescence [3,4]. During the 1970s, colistin was discontinued for clinical application as it was associated with neurotoxicity, nephrotoxicity, and other ailments [5,6]. Recently, colistin was reappraised and is being used as a last-line treatment against Gram-negative bacterial infections [6].

The extensive use of antibiotics for treating human infections caused by multidrug-resistant or highly drug-resistant *Enterobacteriacea*e is threatening the efficacy of colistin [7]. Furthermore, this has led to development of colistin resistance mediated by the transposable and plasmid-borne *mcr* genes that have been reported worldwide in *Enterobacteriaceae* from both humans and food-producing animals’ samples. *Salmonella enteric* serovar infantis is one of the leading serovars among the top five *Salmonella* serovars involved in human infections in Europe [8]. It is most frequently detected in broilers (45.6%) and broiler meat (47.4%), as compared with other meats, which may be complicated by the substantial spread of multi-drug resistant (MDR) strains and extended spectrum beta-lactamase (ESBL)-producing *S. infantis* infections. According to recent reports in Switzerland and the United States, the presence of a conjugative pESI (plasmid emerging from *Salmonella infantis*)-like mega plasmid(harbour the *mcr-1* gene) was found to be a significant cause of this infection, as also reported earlier in Israel and Italy in 2014 and 2015, respectively [9,10,11,12]. The *mcr-1* gene was found in mussels while isolating *Salmonella enteric* serovar Rissen ad ST-469 in northwest Spain during 2012–2016 [13]. This review highlights the uses of colistin consumption in animal-based food, its negative effects, and different approaches and advancements used for detecting colistin in animal-based food.

## 2. Colistin Use in Veterinary Medicine

**Figure 2:**
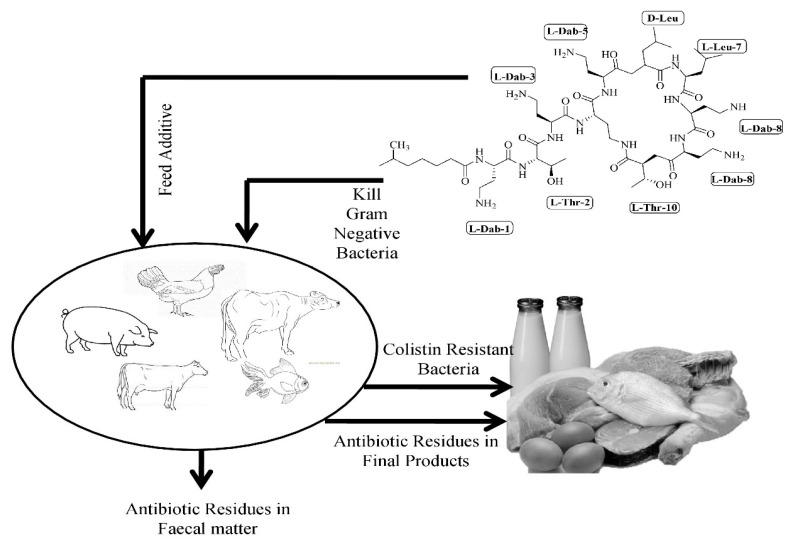
Graphical illustration of the spread of antibiotic-resistant bacteria as well as accumulation of colistin in animal products.

For decades, colistin has been used as an additive in livestock feed for promoting growth and treating intestinal infections [14,15] as shown in Figure 2.

The use of colistin in animal feed and hence human consumption through the food chain has been documented in low and middle-income countries. According to a statistical analysis from 2000 to 2010, Brazil, China, India, Russia, and South Africa account for 13% of colistin use [16]. China is a lead consumer of colistin globally, and about 2875 metric tons of colistin was consumed annually from 2011 to 2015 in this country [17]. In 2006, the European Union forbade the use of colistin in animal food to promote growth [18]. However, colistin continued to be the fifth most highly consumed drug in 2013–2015 in Europe for the treatment purpose as per the European Surveillance of Veterinary Antimicrobial Consumption Report, although no colistin drugs were marketed in Finland, Norway, or Iceland [19,20]. Additionally, some countries like USA and Canada never approved colistin usage in animal feed [19].

The recommended dosage of colistin varies according to the product and species, with 75,000 IU/kg proposed for poultry and 100,000 IU/kg for other animals like calves, rabbits and pigs; these dosages are consistent to 3.75–5 mg/kg. Colistin can be administrated with complete feed, milk, water, or through injection. Approximately 0.01–0.02% of colistin is administered via milk; meanwhile, 25–50 mg/L is taken up by water. In feed, colistin is mixed in a range of 20–40 M IU/100 g, and 0.2 mg of colistin is injected into 1–3-day old chicks [21].

Colistin presence in poultry occurs during the phase where mild colibacillosis is being treated, as described in earlier reports [22,23,24]. Its bioavailability following oral administration is very low as it does not get well absorbed from the gastrointestinal tract [25,26]. Common signs of colibacillosis in poultry are localized (e.g., omphalitis) or systemic (e.g., colisepticemia) that cannot be treated at the attained blood and tissue levels [27]. Drugs such as sulfonamides, tetracycline, and penicillin are more appropriate for use than administering colistin for at least seven days and at higher doses for treating mild colibacillosis [28]. Colistin is effectively used for primary diarrheal disease caused by *Escherichia coli*, which is rarely found in poultry but successfully used as a growth promoter [27]. Various agencies have defined the maximum residue limits (MRL) of colistin in animal-based food, as shown in Table 1.

## 3. Dosing Regimen of Colistin in Animals

Colistin use varies by the type of livestock used as animal-based food. Milk-fed calves were injected with 5 mg/kg of colistin sulfate (CS) (commercially available form), and 16μg/mL was recorded in peak serum concentration analysis. On the other hand, 1.3 L/kg of colistin was used for volume distribution and 3.4 mL/min/kg for renal clearance with an excretion half-life of 5–6 h [34,35]. The serum of dairy cows and calves showed the persistence of colistimethate sodium when it was injected intramuscularly. The highest value (60 IU/mL) of colistin concentration in the serum of cows was recorded within 3 h after the administration of colistin. The highest peak of serum concentration in calves was recorded 1–2 h after colistin administration, and the calculated half-life was found to be two-fold more in cows as compared to calves. The concentration of colistin is very low in milk and sometimes is found to be in the detectable range after a second milking, whereas the well-diffused microbiological method shows no residue in calves [36]. Another study revealed that peak colistin concentration can be measured within 2 h of dosing in serum and traces of it can be measured up to 6 h after intravenous administration, as the detectable range was found to be 0.1–1 μg/mL in serum. There is no detectable limit for oral administration of colistin [37]. The administration of colistimethate sodium intramuscularly increases serum concentration as compared to colistin sulfate for dosages of 3.5 and 7.5 mg/kg, respectively. This further reveals that serum protein has a binding affinity towards colistin sulfate in comparison to colistimethate in ewes [38]. Similar results have also been recorded for dogs [39]. The colistin binding with plasma protein was found to be 40% for cattle. In chickens, after oral injection of 50 mg/kg of colistin, the maximum concentrations of 5.7 and 10.2 μg/mL, respectively, were detected in bile and serum after 2 h.

Sato et al. [40] also conducted experiments on pigs using two different doses of colistin, i.e., 25 and 50 mg/kg. They reported peak serum concentrations of 1.0 and 8.3μg/mL after 1 h of administration of colistin in two different doses, as the sample is untraceable in later stages. The maximum concentrations of 4.0 and 1.0 μg/mL in bile and serum of pigs were detected respectively after oral administration of 25 mg/kg of colistin [40]. Another study reported the intractability of colistin in the serum of gnotobiotic piglets which were fed 40 mg/kg of colistin in sterilized milk [41]. The research was conducted on pigs to assess the effect of oral dosages of 2.5 and 5.0 mg/kg along with a 2.5 mg/kg intravenous dose, and it was observed that the peak concentration of plasma was attained after 30 min of administration and the half-life for both the doses was found to be 4.5 h with a clearance rate of about 3 mL/kg/min [42]. CS concentrations were very difficult to calculate in the plasma of the healthy pigs after oral administration, despite the use of exact and accurate analytical methods [43,44]. A concurrent oral challenge of pigs with an *Enterotoxigenic Escherichia coli* (ETEC) was done. The F4 strain did not increase CS intestinal absorption in a subclinical induction model of post-weaning diarrhoea (PWD) [44]. However, CS concentrations in plasma were higher in pigs with clinical post-weaning diarrhoea following an experimental oral challenge as compared to the unchallenged pigs [45]. These studies revealed the low absorption of CS through the gastro-intestinal tract of pigs even in infected animals and corroborate the involvement of oral CS administration in increasing colistin resistance by exerting selection pressure (due to antibiotic) on the intestinal flora of pigs [46].

## 4. Negative Consequences of Colistin Consumption

Until 2015, colistin resistance in the *Enterobacteriaceae* family was believed to be generated via chromosomal mechanisms which modified the lipopolysaccharide (LPS) layer by adding 2-aminoethanol, phosphoethanolamine (PetN) (a derivative of 2-aminoethanol), or other efflux pumps or by forming capsules in these microorganisms [47]. Lipid A modification of LPS can be associated with mutations triggering the activation of two-component systems including PmrA/PmrB and PhoP/PhoQ or inactivating the *mgrB* gene, which induces negative feedback of the PhoP/PhoQ system in Gram-negative species. In *Escherichia coli,* the *etk* and *mgr R* genes have been found to confer resistance against colistin [48]. In 2015, Chinese researchers studied the colistin-resistant strains of bacteria and reported the presence of the *mcr*-1 gene, which can transfer itself from one bacterial strain to another [49]. The *mcr*-1 gene encodes a phosphoethanolamine transferase, which catalyzes the addition of phosphoethanolamine (a cationic molecule) to lipid A of LPS, which changes the charge of the cell membrane, and as result colistin (cationic) is unable to bind and triggers the lysis of the cell membrane [50]. The PCR-based screening has enabled researchers to find *mcr*-2 to *mcr*-8 genes (plasmid-mediated colistin-resistant genes) and revealed theprevalence of the *mcr*-2 gene in *Escherichia coli* strains isolated from bovine and porcine [51,52,53,54,55,56,57]. Moreover, *mcr*-2 to *mcr*-8 genes share 44–77% similarity with *mcr*-1, and the gene products synthesized by them have 32–83%similarity to the amino acid sequence of *mcr*-1. A list of plasmid-borne *mcr*-1 in bacteria isolated from animal-based food is shown in Table 2.

Colistin is an ancient drug that was banned because of its nephrotoxicity and neurotoxicity activity in humans; however, it was reintroduced to treat carbapenem resistance in Gram-negative bacteria (Supplementary Table S1). Unfortunately, colistin resistance mechanisms have now been documented in *Enterobacteriaceae* strains capable of producing carbapenemase, making them resistant to both classes of drugs and a global health concern [76,77,78,79].

In 2016, the government of China banned the use of colistin as a food additive for livestock. China alone was using 8000 tons of colistin per annum, whereas global production was 12,000 tons per annum. Despite this ban, agrichemical companies in China were the leading colistin producer and tons of colistin were exported to countries like India, South Korea, and Vietnam [80]. In India, five animal pharmaceutical companies advertise products containing colistin for promoting growth or use for metaphylactic purposes. As per the investigation carried out by the Bureau of Investigative Journalism of London, chickens raised in India are heavily dosed with strong antibiotics. Venky’s, the chief supplier of chicken products in India, has been reported to use the antibiotic colistin for therapeutic purposes [81]. These practices are highly unsafe as drug-resistance is very common, and about 57% of Gram-negative bacteria in India are carbapenem-resistant. Therefore, India depends on colistin for treating acute infections in humans (in contrast, resistance to carbapenem in *Klebsiella pneumoniae* is less than 1% in the United Kingdom) [80].

The Government of India did take the initiative to ban the usage of colistin antibiotics as a growth supplement, but this initiative has not yet been associated with any regulatory body. Now the Food Safety and Standards Authority of India (FSSAI) claims to have fixed the tolerance level of antibiotics in food-based items, and they have also revised current standards governing toxics, residues, and contaminants under the 2011 regulations [82]. Finally, in 2019, the FSSAI implemented a complete ban on colistin use in India [83].

## 5. Routine Methods for Colistin Detection in Animals and Its Associated Challenges

Colistin, being a polar drug, forms a strong bond with phospholipids or proteins, which makes drug extraction a more complicated process in tissues [84]. Hence, limited systems have been created and are available to find colistin antibiotics in food, as shown in Table 3.

Earlier, this drug was identified by adding chromophore/fluorophore groups which allow its detection with conventional LC detectors. Sin et al. [85] first published a paper for bacitracin and colistin detection using LC–MS/MS in the kidney, liver, and milk. Deproteinized milk samples were extracted with a mixture of trichloroacetic/formic acid and the presence of bacitracin and colistin in extracts was determined using a reversed-phase Alltima BDS C_18_ column using a gradient elution of ammonium formate buffer and 0.1% formic acid in acetonitrile at 0.2 mL min^−1^. For identification and quantification of major components of these two polypeptides, electrospray LC–MS/MS with time scheduled multiple reaction monitoring (MRM) based upon the intensities of mass fragments from the bacitracin A at 712→199 amu and 712→227 amu and colistin A at 586→101 amu, 586→202 amu and 586→241 amu were used.

An upgraded procedure for the detection of colistin B in the liver, muscle, and milk was also developed [96]. This method proved to be a fast screening and quantitative protocol for monitoring the concerned polypeptides present in food as a part of a surveillance program. Xu et al. [86] developed an analytical procedure for colistin A and B in fish products. In this study, the extraction of samples was done with 1.0 mol/L of hydrochloric acid (HCl) in methanol–water, and the sample was further purified on PLS solid-phase extraction columns. Multiple reaction monitoring was performed afterward using precursor–product ion combinations and resulted in mean recovery between 72.9% and 82.9%. Kaufmann and Widmer [97] also reported a multi-residue method capable of detecting five polymyxins with selective and acceptable recoveries for all compounds. In this study, using a modern core-shell column with an eluent with trifluoroacetic acid, formic acid and acetonitrile resulted in chromatographically well-resolved analyte peaks. Boison et al. [94] further improved this technique and were able to detect seven polymyxins in chicken muscle. This process does not use ion-pairing reagents during the mobile phase, which permits the use of the same instrument again to perform different analyses, whereas the use of ion-pairing reagents requires effective washing/cleaning of LC lines, which may lead to instrument downtime and damage with trifluoroacetic acid (TFA) before switching the instrument for the analysis of other samples. All of the above methods follow the same treatment strategy, i.e., an acid extraction protocol involving acetonitrile or methanol or water in different proportions and subjected to reversed-phase SPE (solid-phase extraction) to lower the aggregates of intrusive substances.

Saluti et al. [95] created a novel system for quantification as well as identification of twelve aminoglycosides (AGs) and two colistins in bovine meat and milk through liquid chromatography combined with quadrupole-orbitrap mass spectrometry and hydrophilic interaction liquid chromatography (HILIC). In HILIC, bare silica poroshell 120 showed the optimum result and the recoveries of all the drugs were near 72–87% in meat (except colistins) and 82–96% in milk. In another study, an efficient analytical system was created for the simultaneous determination of seven cyclopolypeptide antibiotics (vancomycin, polymyxin B, polymyxin E, teicoplanin A2, cacitracin A, daptomycin, and virginiamycin M1) using liquid chromatography–tandem mass spectrometry [93].

**Figure 3:**
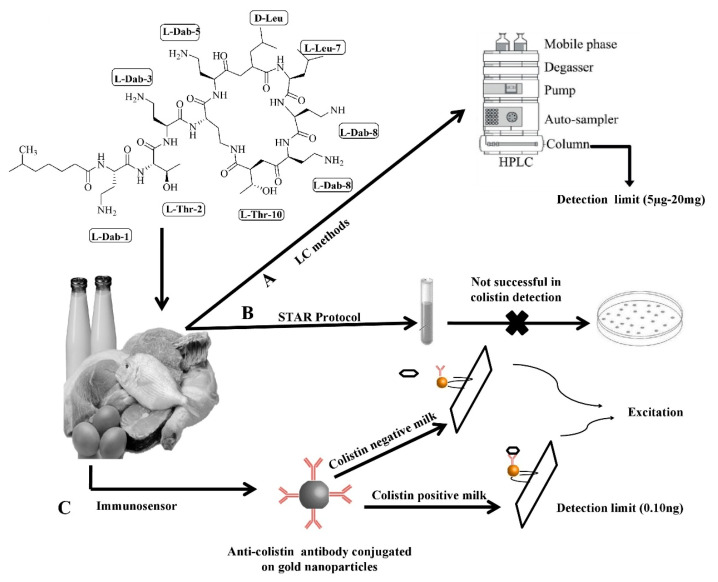
Graphical illustration of different methods used for colistin detection in animal-based food.

LC–MS/MS and HPLC have enabled researchers to precisely identify colistin from biological entities, but it requires skilled workforce and massive sample pre-treatment, involving both solid-phase extraction and protein precipitation as shown in Figure 3.

These techniques are mainly used for laboratory examination and are not employed for robust screening of bulky samples.

A microbiological technique, i.e., screening test for antibiotic residues (STAR), was developed to analyze the milk samples spiked with eight different concentrations of colistin according to the sensitivity of bacterial strains against this antibiotic. The detection limit of this approach was found to be 1 mg/L. During authentication of the STAR protocol, the reading of colistin in milk was measured to be 200–2000-fold more than its maximum residue limit (50 µgL^−1^), thus leading to rejection of this protocol for colistin detection [98,99].

## 6. Conclusions and Future Outlook

Colistin has been identified as an imperative alternative for MDR Gram-negative microbes. However, the emergence of colistin-resistant strains has created the havok as it is the last resort for treating infection. Moreover, many reports have linked the colistin resistance with inadequate dosing. The challenges have made us realize the importance of optimized dosage, exclusively in chronically ill patients with MDR strains. The resistant strains of colistin remain the matter of great concern and make it of utmost importance to detect the colistin in food animals. However, there are very few conventional methods available for the detection of colistin use in animal-based food and other livestock. Most of these methods can detect colistin up to a sensitivity limit. The laboratory check-ups to assess the effective use of colistin treatment at farms need to be highlighted by veterinarians. The data on the usage of colistin in animal-based food are of vital necessity, as it supplies a base for the evolution of national policies and also elucidates the hazards of colistin resistance management and evaluates the effect of possible involvement [100,101].

Recently, a surface-enhanced Raman scattering (SERS) immune-sensor was developed for the detection of colistin in milk [15]. In this method, 5,5-dithiobis-2-nitrobenzoic acid (DTNB) was labeled on gold nanoparticles along with anti-colistin monoclonal antibody (mAb). The SERS immune-sensor was attached to the lateral flow strip, which was further attached with Raman signal readout to quantify the colistin amount with high precision. This method can detect concentrations as low as 0.10 ng/mL colistin in milk, which is higher than the value obtained earlier using ELISA and also the maximum residue limit determined by the European Union. Additionally, the spiking experiments displayed a high accuracy of the SERS immune-sensor, with a recovery of 88.1–112.7% with a standard deviation of less than 15%. This approach has an advantage in terms of robustness and time of detection (below 20 min) over the conventional techniques.

It is wellknown that there is dire need for improvement in colistin detection with high accuracy and specificity in animal food. Few bio-sensors have been developed for identifying food-related disorders, like the transglutaminase-based nano-sensor for the prognosis of the celiac disorder, as well as human pathogens, e.g., quick detection of *Streptococcus pyogenes* and *Leptospirainterrogans* [102,103,104]. New improvements and novel changes are required in techniques based on precision and specificity to fulfil the future demand for colistin detection and to develop novel biosensors for rapid identification of colistin in animal-based food.

## Figures and Tables

**Table 1:** Maximum residue limits (MRLs) of colistin recommended in animal-based food by different regulatory agencies.

Animal Species	Target Tissue	MRLs (Per Kg)	Reference
All food producing animal spp.	Fat, muscle, liver	150 µg, 150µg, 150 µg	[29]
All food producing animal spp.	Kidney	200 µg	[29]
All food producing animal spp.	Milk	50 µg	[29]
All food producing animal spp.	Eggs	300 µg	[29]
Cattle, sheep’s	Fat, muscle, kidney, liver, milk	150 µg, 150 µg, 200 µg, 150 µg, 50 µg	[30]
Pig, goat, rabbit	Fat, muscle, liver, kidney	150 µg, 150 µg, 150 µg, 200 µg	[30]
Chicken	Fat, liver, kidney, eggs	150 µg, 150 µg, 200 µg, 300 µg	[30]
Turkey	Fat, muscle, liver, kidney	150 µg, 150 µg, 150 µg, 200 µg	[30]
Cattle, lamb	Milk	50 µg	[31]
Cattle, lamb, swine, chicken, rabbit	Fat, muscle, liver, kidney	150 µg, 150 µg, 150 µg, 200 µg	[31]
Bovine	Muscle, liver, kidney	150 µg, 150 µg, 200 µg	[32]
Porcine	Liver, kidney	150 µg, 200 µg	[32]
Poultry	Muscle, liver, kidney	150 µg, 150 µg, 200 µg	[32]
Pig, cattle, chicken	Muscle, fat, liver	150 µg	[33]
Cattle	Milk	50 µg	[33]
Pig, cattle, chicken	Kidney	200 µg	[33]

**Table 2:** Isolation of colistin-resistant microbes from animal-based food in different countries.

Country	Type of Animals	Type of Samples	Sample Size	Type of Microbes	Detection Basis	Reference
Nepal	Healthy chickens	Cloacal swabs	324	*Escherichia coli*	*mcr-*1	[58]
China	Healthy chickens	Lung, spleen	644	*Escherichia coli*	*mcr-*1	[59]
	Pigs	Liver	113			
	Cows	Milk	61			
	Ducks	Liver	44			
Iran	Healthy broilers	Cloacal swabs	503	*Klebsiella pneumoniae*	*mcr-*1, 2, 3, 4	[60]
	Dead broilers		388			
	Dead lying hens		30			
	Dead turkeys		23			
Vietnam	Healthy chickens	Cloacal swabs	NS	*Escherichia coli*	*mcr-*1	[61]
	Pigs					
Brazil	Healthy chickens	Breast	20	*Escherichia coli*	*mcr-*1	[62]
		Thigh	20			
		Liver	1			
Denmark	Chicken meat	ND	NS	*Escherichia coli*	*mcr-*1	[63]
Spain	Swine	Lymph node	NS	*Salmonella enterica, Escherichia coli*	*mcr-*1	[64]
		Faeces				
	Turkey	Faeces	NS	*Escherichia coli*		
Switzerland	Chicken meat	ND	6	*Escherichia coli*	*mcr-*1	[65]
Germany	Healthy chicken	Drumsticks	500	*Cirobacter freundii, Klebsiella oxytoca, Pantoea agglomerans*	Disc diffusion	[66]
	Pork	Belly	500	*Escherichia coli, Klebsiella oxytoca*		
Japan	Diseased swine	ND	NS	*Escherichia coli*	*mcr-*1	[67]
Algeria	Healthy chickens	ND	NS	*Escherichia coli*	Disc diffusion	[68]
Taiwan	Diseased Chickens	ND	450	*Salmonella* spp.	*mcr-*1	[69]
	Pigs		279			
	Ducks		206			
	Turkeys		170			
	Geese		88			
Great Britain	Diseased pigs	Small intestine	3	*Escherichia coli, Salmonella typhimurium*	*mcr-*1	[70]
Great Britain	Healthy Pigs	Cecums	2509	*Escherichia coli*	Disc diffusion	[71]
	Cattle	Distal rectums	891			
	Sheep	Distal rectums	973			
Italy	Diseased pigs	Rectal swabs, faeces, intestines	NS	*Escherichia coli*	Disc diffusion, *mcr-*1	[72]
Great Britain	Healthy pigs	Cecal contents	NS	*Moraxella* spp.	MIC and *mcr-*1,2	[52]
France	Diseased pigs	Intestinal	63	*Escherichia coli*	Disc diffusion	[48]
		Septicemia	2			
		Nervous system	1			
		Lymph node	1			
		Urine	1			
Botswana	Beef	Meat cubes	134	*Escherichia coli* O157: H7	Disc diffusion	[73]
		Minced meat	133			
		Fresh sausages	133			
India	Poultry	ND	NS	*Salmonella* spp.	Disc diffusion	[74]
India	Chickens	Faecal, cecal	434	*Salmonella enterica*	Disc diffusion	[75]
	Ducks	Faecal	38			
	Emus	Faecal	35			

ND—not defined; NS—not specified.

**Table 3:** Different conventional methods used for the detection of colistin in animal-based food.

Country	Sample	Method Used	Chromatography Conditions Used				Detection Limit	Reference
			Model	Column	Solvent	Flow Rate		
China	Spiked bovine milk	HPLC–MS/MS	An HPLC (Hewlett-Packard HP 1100 series, Rockville, MD, USA) integrated system consisting of a 100-well auto-sampler, a 100 µL sample loop, a degasser, a quaternary pump and a thermostated column oven set at 25 °C was used	Chromatographic separation was performed in a 250 mm × 2.1 mm, 5 µm Alltima C_18_ separation column (Alltech, Deerfield, MA, USA) and a corresponding C_18_ guard column (7.5 mm × 4.6 mm)	Mobile phase A: 0.1% formic acid in acetonitrile and mobile phase B: saturated ammonium formate:formic acid:acetonitrile:water (1:5:50:950, *v*/*v*/*v*/*v*)	Flow rate of 0.2 mL min^−1^ under a gradient elution program comprised of two mobile phases	50 µg/Kg	[85]
China	Spiked fishery products	UPLC–MS/MS	A UPLC–MS/MS system comprised an Acquity UPLC system connected online with a Quattro Premier tandem mass spectrometer (Waters, Milford, MA, USA)	The column used was an ACQUITYTM BEH C_18_ reversed phase column (2.1 mm × 100 mm, 1.7 µm particle size) maintained at 40 °C	Mobile phase was 0.2% formic acid in acetonitrile and 0.2% formic acid in water	Flow rate and temperature of the drying gas (N_2_) were 750 L h^−1^ and 350 °C, respectively. The cone gas flow (N_2_) was 50 L h^−1^	10 µg/Kg (colistin A), 40 µg/Kg (colistin B)	[86]
Hungary	Spiked pig feeds	HPLC–fluorescence detector	JASCO PU-980 high pressure pump (JASCO, Kyoto, Japan)	A TSK ODS 120T column (150 × 4.6 mmID, 5 µm) was used with an injection volume of 25 µL	Mobile phase was 22:78 *v/v* acetonitrile–50 mM sodium sulfate, 20 mM orthophosphoric acid, 25 mM triethylamine	Flow rate of the mobile phase and post-column reagent were 1.5 and 1.0 mL min^−1^, respectively	20 mg/Kg	[87]
Spain	Spiked animal feeds	HPLC–fluorescence detector	Thermo HPLC system equipped with a P200 gradient pump	Analytical column (150 × 4.6 mm i.d.) used was packed with Ultracarb 5 µm ODS 30%C. Guard columns (50 × 4.6 mm i.d.) were packed with dry 40 µm Pelliguard LC-18	Mobile phases with methanol and acetonitile	Flow of 1.5 mL	5 mg/Kg	[88]
France	Spiked bovine milk and tissues (muscle, liver, kidney, fat)	HPLC–MS	The HPLC system consisted of a solvent delivery pump (model P2000, Thermo Separation Products, Les Ulis, France), an injection valve (model 7725i, Rheodyne, Cotati, CA, USA)	An analytical column (125 × 4 mm i.d.) pre-packed with 5 μmNucleosil C_18_ (Macherey-Nagel, Düren, Germany)	Mobile phase was acetonitrile and a 0.035 M triethylamine solution adjusted to pH 2.5 with phosphoric acid and mixed in 17:83 (*v*/*v*) proportions	The flow rate was 1.5 mL/min	25 µg/L (milk), 100 µg/Kg (tissues)	[89]
China	Swine liver, chicken eggs, feed, swine muscles, chicken muscles, bovine muscles, sheep muscles, bovine raw milk	UHPLC–MS/MS	An Acquity ultra-performance liquid chromatography system (Waters, Milford, MA, USA)	An Acquity BEH C_18_ column (50 mm × 2.1 mm i.d., 1.7 μm particle size) (Waters, Milford, MA, USA)	Mobile phases comprised of 0.5% formic acid in water (solvent A) and 0.5% formic acid in acetonitrile (solvent B)	Flow rate was 0.4 mL/min with the following gradient program: 0–0.5 min, 95% A; 0.5–3.0 min, 95–50% A; 3.0–4.0 min, 50–5% A; 4.0–4.1 min, 5–95% A; 4.1–5.5 min, 95% A	5–30 µg/Kg	[90]
Belgium	Spiked swine manure	UHPLC–MS/MS	An Acquity UPLC H-class system (Waters, Milford, MA, USA)	Reversed-phase Kinetex C_18_ column (100 mm × 2.1 mm i.d., 1.7 µm) with a SecurityGuard Ultra guard cartridge system (Phenomenex, Utrecht, The Netherlands)	The elution was performed gradually with changing amounts of H_2_O/MeCN (95/5) + 0.5% FA + 0.1% ammonium formate (solvent A) and MeCN + 0.1% FA (solvent B). The gradient (15 min) was initiated with 95% of solvent A (0–1 min), followed by a linear decrease of A to 75% (1–3 min). From min 3–5, there was a linear decrease of solvent A to 0% and this was held until min 7. Re-equilibration of the gradient at 95% A was held from min 7–15	Flow at 400 µL/min	20.2 µg/Kg (colistin A), 15 µg/Kg (colistin B)	[91]
China	Spiked swine and poultry feeds	UHPLC–MS/MS	LC–MS/MS system (Thermo Electron Corp., Wyman, Waltham, MA, USA) consisting of a Finnigan Surveyor Plus system with an online degasser, a Surveyor autosampler and a TSQ Quantum triple quadrupole mass spectrometer equipped with an electrospray interface operating in the positive mode (ESI+)	Separation was performed on 150 mm × 2.1 mm, 5 μm Hypersil Gold C_18_ analytical columns (Thermo Electron Corporation, Waltham, MA, USA)	Mobile phase A consist formic acid in water and mobile phase B formic acid in ACN	Flow-rate of 0.2 mL min^−1^	27.5 µg/Kg (colistin A), 25.7 µg/Kg (colistin B)	[92]
China	Spiked piglet premix, pig feed additive, poultry complete feed, pig complete feed and fattening pig premix	UHPLC–MS/MS	Shimadzu liquid chromatography system (Shimadzu, Kyoto, Japan)	Separations were carried out on a Phenomenex Kinetex Biphenyl column (50 mm × 2.1 mm i.d., 2.6 µm particle size, Phenomenex, Torrance, CA, USA)	Mobile phase consisted of 0.1% FA in ACN solution (A) and 0.1% FA in water solution (B) with the following gradient elution program: 0 min, 6% A; 2 min, 6% A; 5 min, 40% A; 14 min, 70% A; 14.1 min, 6% A; 18 min, 6% A	Flow rate of 0.2 mL/min.	5–20 µg/Kg (colistin A), and (colistin B)	[93]
Canada	Spiked chicken muscle	UPLC–MS/MS	Waters Acquity UPLC interfaced to a Waters Micromass triple quadrupole Premier mass spectrometer equipped with an ESI source and controlled by MassLynx 4.1 software(Waters, Milford, MA, USA)	Poroshell 120, 100 × 2.1 mm id, 2.7 μm (Agilent Technologies, Mississauga, ON Canada)	Mobile phase A (0.1% formic acid in water)and mobile phase B (methanol)	Flow rate of 0.40 mL/min	39 µg/Kg (colistin A), 50 µg/Kg (colistin B)	[94]
Italy	Spiked bovine milk, meat	HPLC–MS	Thermo Ultimate 3000 High Performance Liquid Chromatography system (Thermo Scientific, San Jose, CA, USA)	InfinityLab Poroshell 120 HILIC column (100 × 2.1 mm; 2.7 μm, Agilent Technologies, Santa Clara, CA, USA) connected with the InfinityLab Poroshell 120 HILIC guard column (5 × 2.1 mm, 2.7 μm)	Eluent A was an aqueous solution containing 1% (*v*/*v*) formic acid (FA) and 1 mM ammonium formate (AF), eluent B was acetonitrile. The gradient was initiated with 20% eluent A for 2 min, continued with linear increase to 35% A in 5 min. In 1 min eluent A increased to 95% and this condition was maintained for 7 min. The system returned to 20% B in 0.1 min and was re-equilibrated for 4 min (run time: 17 min)	Flow rate was 0.25 mL min^−1^	33 µg/Kg	[95]
Hong Kong	Spiked bovine milk and tissues	HPLC–MS/MS	An integrated HPLC system (Hewlett–Packard HP 1100series, Rockville, MD, USA) consisting of a 100-well autosampler, a degasser, two-channel binary pump, and atemperature control oven (set at 25 °C), and interfaced with a TSQ Quantum Discovery mass spectrometer (Thermo-Finnigan, San Jose, CA, USA)	150 mm×2.1 mm, 5 μm Phenomenex Luna C_18_ analytical column (Torrance, CA, USA) connected to a 7.5 mm × 4.6 mm Alltech Alltima C_18_ guard column (Deerfield, IL, USA)	Mobile phases, which were comprised of a mixture of (A) 0.1% formic acid in water and (B) 0.1% formic acid in acetonitrile, were delivered under a gradient elution program (0–4 min: 95% A, 5% B; 4–8 min: 30% A, 70% B and held for 4 min; 12 min: 95% A, 5% B and held for 3 min to restore initial conditions before the next injection	Flow-rate of 0.25 mL min^−1^	1–16 µg/Kg (colistin A), 6–14 µg/Kg (colistin B)	[96]
Switzerland	Spiked bovine liver, kidney, muscle, egg, milk	UHPLC–MS/MS	Acquity system (sample and solvent manager) from Waters (Millford, MA, USA)	Kinetex C_18_, 2.1 × 150 mm × 2.6 µm column with an installed pre-filter (Krud-katcher), both from Phenomenex (Torrance CA, USA)	Mobile phase A: 50 mL acetonitrile, 3 mL of formic acid and 0.1 mL of trifluoroacetic acid were transferred into a 1000 mL volumetricflask and diluted to volume with purified water; Mobile phase B: 50 mL of purified water, 3 mL of formic acidand 0.1 mL of trifluoroacetic acid were transferred into a 1000-mLvolumetric flask and diluted to volume with ACN	Linear gradient was used: 0–2 min with 8% B and flow0.4 mL min^−1^, 2–7 min with 8–20% B, 7–8 min with 20–30% B, 8–11 min with 30–100% B, 11–11.1 min with 100% B and flow 0.4–0.8 mL min^−1^, 11.1–12.5 min with 100%, 12.5–12.51 min with 100–8% B and flow 0.8–0.4 mL min^−1^. 12.51–14 min with 8% B and flow 0.4 mL min^−1^	Muscle 15 µg/Kg (colistin A), 30 µg/Kg (colistin B); kidney 30 µg/Kg (colistin A), 30 µg/Kg (colistin B); liver 30 µg/Kg (colistin A), 30 µg/Kg (colistin B); egg 20 µg/Kg (colistin A), 30 µg/Kg (colistin B); milk 20 µg/Kg (colistin A), 40 µg/Kg (colistin B);	[97]
France	Spiked milk	Disc diffusion method (STAR protocol)	NA	NA	NA	NA	1 mg/L	[98]

NA—not applicable.

## Supplementary Materials

The following are available online at https://www.mdpi.com/2076-2615/10/10/1892/s1, Table S1. Antibiogram pattern of pathogenic microbes against colisitn isolated from patients.
