# Supplementary material for: Understanding of Colistin Usage in Food Animals and Available Detection Techniques: A Review
Source: Animals (Basel). 2020 Oct 16;10(10):1892. doi: 10.3390/ani10101892 (PMC7602861; doi:10.3390/ani10101892)
Supplement: Supplementary file 1 [file animals-10-01892-s001.pdf]

Table S1. Antibiogram pattern of pathogenic microbes against colisitn isolated from patients

Types of sample	Sample Size	Types of Microbes	Types of Test	Pattern	Reference
Sputum; nasal; wound; blood; urine; bronchoalveolar lavage	16	*Acintobacter baumannii*	MIC	Resistant (14)	[1]
Blood; bronchial aspirates; cerebrospinal fluids; carriage; wound infections; skin ulcers; catheters; surgical drains; conjunctival exudates; nasal exudates; peritoneal fluid; intraabdominal abscess	115	*Acintobacter baumannii*	MIC by E-test	Resistant (22)	[2]
Clinical specimens	44	*Acinetobacter* spp.	MIC	Susceptible (44)	[3]
Clinical specimens	265	*Acinetobacter* spp.	MIC	Resistant (48)	[4]
Clinical specimens	250, 40, 226, 124,36	*Klebsiella pneumonia, Enterobacter aerrogenes, Enterobacter* spp., *Acintobacter baumannii, Pseudomonas aeruginosa Stentorophomonas maltophilia*	MIC by E-test	Susceptible (187), (33), (216), (115), (41)	[5]
Cystic fibrosis	23	*Pseudomonas aeruginosa*	MIC	Resistant (13)	[6]
Clinical specimens	18, 13, 8, 16, 33, 17	*Acinetobacter* spp., *Escherichia coli, Enterobacte* spp., *Klebsiella pneumonia, Stentorophomonas maltophilia*	MIC	Susceptible (18), (18), (6), (15), (22), (0)	[7]
Clinical specimens	21	*Klebsiella pneumonia*	MIC	Susceptible (15)	[8]
Bacteremia	2	*Escherichia coli*	MIC	Resistant (2)	[9]
Clinical specimens	9	*Escherichia coli*	MIC	Resistant (9)	[10]
Clinical specimens	16533, 7446, 9786	*Enterobacteriaceae, Acinetobacter* spp., *Pseudomonas* spp.	MIC by E-test	Susceptible (15374), (7344), (9701)	[11]
Respiratory secretions; wound swabs; blood; urine; body fluid	250	*Acinetobacter* spp.	MIC by E-test	Resistant (30)	[12]
Diarrhea	1, 1	*Klebsiella pneumonia, Escherichia coli*	MIC	Resistant (1), (1)	[13]
Bloodstream infections	1495, 571	*Escherichia coli, Klebsiella pneumonia*	MIC	Resistant (20), (1)	[14]
Lower respiratory tract infections	58, 19, 13, 8, 7, 4	*Acinetobacter* spp., *Pseudomonas aeruginosa, Klebsiella pneumonia,*	Disc diffusion	Resistant (3), (0), (0), (0), (0), (0)	[15]

	Escherichia coli, *Citrobacter* spp., *Enterobacter* spp.	

References

1. Li, J.; Rayner, C.R.; Nation, R.L.; Owen, R.J.; Spelman, D.; Tan, K.E.; Liolios, L. **Heteroresistance** to **colistin** in **multidrug-resistant *Acinetobacter baumannii***. *Antimicrob Agents Chemother*. 2006, *50*, 2946-2950.
2. Arroyo, L.A.; García-Curiel, A.; Pachón-Ibañez, M.E.; Llanos, A.C.; Ruiz. M.; Pachón, J.; Aznar, J. Reliability of the E-Test Method for Detection of Colistin Resistance in Clinical Isolates of *Acinetobacter baumannii*. *J Clin Microbiol*. 2005, *43*, 903-905.
3. Tan, T.Y.; Ng, L.S.; Poh, K. Susceptibility testing of unconventional antibiotics against multiresistant *Acinetobacter* spp. by agar dilution and Vitek 2. *Diagn Microbiol Infect Dis*. 2007, *58*, 357-361.
4. Ko, K.S.; Suh, J.Y.; Kwon, K.T.; Jung, S.I.; Park, K.H.; Kang, C.I.; Chung, D.R.; Peck, K.R.; Song, J.H. High rates of resistance to colistin and polymyxin B in subgroups of *Acinetobacter baumannii* isolates from Korea. *J Antimicrob Chemother*. 2007, *60*, 63-1167.
5. Galani, I.; Kontopidou, F.; Souli, M.; Rekatsina, P.D.; Koratzanis, E.; Deliolanis, J.; Giamarellou, H. Colistin susceptibility testing by Etest and disk diffusion methods. *Int J Antimicrob Agents*. 2008, *31*, 434-439.
6. Li, J.; Turnidge, J.; Milne, R.; Nation, R.L.; Coulthard, K. In **vitro pharmacodynamic properties** of **colistin** and **colistin methanesulfonate against *Pseudomonas aeruginosa* isolates** from **patients** with **cystic fibrosis**. *Antimicrob Agents Chemother*. 2001, *45*, 781-785.
7. Tan, T.Y.; Ng, S.Y. The **in-vitro activity** of **colistin** in **gram-negative bacteria**. *Singapore Med J*. 2006, *47*, 621-624.
8. Poudyal, A.; Howden, B.P.; Bell, J.M.; Gao, W.; Owen, R.J.; Turnidge, J.D.; Nation, R.L.; Li, J. **In vitro pharmacodynamics** of **colistin against multidrug-resistant *Klebsiella pneumonia*.** *J Antimicrob Chemother*. 2008, *62*, 1311-1318.
9. Nordmann, P.; Lienhard, R.; Kieffer, N.; Clerc, O.; Poirel, L. **Plasmid-mediated colistin-resistant *Escherichia coli*** in **bacteremia** in **Switzerland**. *Clin Infect Dis*. 2016, *62*, 1322-1323.
10. Rapoport, M.; Faccone, D.; Pasteran, F.; Ceriana, P.; Albornoz, E.; Petroni, A.; M.C.R. Group.; Corso, A. First description of *mcr-1*-mediated colistin resistance in human infections caused by *Escherichia coli* in Latin America. *Antimicrob Agents Chemother*. 2016, *60*, 4412-4413.
11. Rossi, F.; Girardello, R.; Cury, A.P.; Di Gioia, T.S.; Almeida, J.N.; Duarte, A.J. Emergence of colistin resistance in the largest university hospital complex of São Paulo, Brazil, over five years. *Braz J Infect Dis*. 2016, *21*, 98-101.
12. Al-Sweih, N.A.; Al-Hubail, M.A.; Rotimi, V.O. Emergence of tigecycline and colistin resistance in *Acinetobacter* species isolated from patients in Kuwait Hospitals. *J Chemother*. 2011, 23, 13-16.
13. Gu, D.X.; Huang, Y.L.; Ma, J.H.; Zhou, H.W.; Fang, Y.; Cai, J.C.; Hu, Y.Y.; Zhang, R. Detection of colistin resistance gene *mcr-1* in hypervirulent *Klebsiella pneumoniae* and *Escherichia coli* isolates from an infant with diarrhea in China. *Antimicrob Agents Chemother*. 2016, *60*, 5099-5100.
14. Quan, J.; Li, X.; Chen, Y.; Jiang, Y.; Zhou, Z.; Zhang, H.; Sun, L.; Ruan, Z.; Feng, Y.; Akova, M.; Yu, Y. Prevalence of *mcr-1* in *Escherichia coli* and *Klebsiella pneumonia* recovered from bloodstream infections in China: a multicentre longitudinal study. *Lancet Infect Dis*. 2017, *17*, 400-410.
15. Bhatta, D.R.; Hamal, D.; Shrestha, R.; Supram, H.S.; Joshi, P.; Nayak, N.; Gokhale, S. Burden of multidrug resistant respiratory pathogens in intensive care units of tertiary care hospital. *Asian J Med Sci*. 2019, *10*, 14-19.
